# Co-expression prognostic-related genes signature base on propofol and sevoflurane anesthesia predict prognosis and immunotherapy response in glioblastoma

**DOI:** 10.1080/07853890.2023.2171109

**Published:** 2023-03-01

**Authors:** Zhiqi Hou, Dexing Luo, Huanhuan Luo, Qiang Hui, Yongqing Xu, Xiaofeng Lin, Zhibin Xu

**Affiliations:** a Hui Zhou Central People’s Hospital, HuiZhou, Guangdong, China; b Department of Anesthesiology, Hui Dong County People’s Hospital, HuiZhou, Guangdong, China; c Department of Basic Medical Sciences, Aspire (Hong Kong) Medical Research Center, Hong Kong, China

**Keywords:** Glioblastoma, sevoflurane, propofol, prognosis, MGMT

## Abstract

**Objectives:**

Anesthetic drugs had been reported may impact the bio-behavior of the tumor. Propofol and sevoflurane are common anesthetics in the operation for glioblastoma (GBM). This study aims to establish a co-expression prognostic-related genes signature base on propofol and sevoflurane anesthesia to predict prognosis and immunotherapy response in GBM.

**Method:**

GPM tissues with different anesthetics gene expression profiles (GSE179004) were obtained from the Gene Expression Omnibus (GEO) database. Core modules and central genes associated with propofol and sevoflurane anesthesia were identified by weighted gene coexpression network analysis (WGCNA) and establish a risk score prognostic model. Immune cell signature analysis in TCGA datasets was predicted via CIBERSORT. At last, serum methylation level of O6-methylguanine-DNA methyltransferase (MGMT) promoter was detected in GPM patient in different time during perioperative period.

**Results:**

The burlywood1 group screened was significantly associated with sevoflurane-treated GBM tissue. 22 independent prognostic differential genes were construct a prognostic-related genes risk score in GBM, and showed good predictive ability. The risk score was strongly correlated with the age of the patients, but not with the sex of the patients. In addition, the differential responses to immunotherapy in high and low risk groups were analyzed, indicating that sevoflurane signature genes were consistent in the classification of gliomas. High-risk patients have high T-cell damage score and are less sensitive to immunotherapy. At last, serum methylation level of MGMT promoter was decreased in GBM patients during propofol and sevoflurane anesthesia.

**Conclusions:**

Propofol and sevoflurane anesthesia associated impact on the gene expression of GBM, included the methylation level of MGMT promoter. Propofol and sevoflurane anesthesia-based risk score prognostic model, which has good prognostic power and is an independent prognostic factor in GBM patients. Therefore, this model can be used as a new biomarker for judging the prognosis of GBM patients.

**KEY MESSAGES**
- Propofol and sevoflurane anesthesia-based risk score prognostic model has good prognostic power and is an independent prognostic factor in GBM patients.
- High Propofol and sevoflurane anesthesia-based risk score GBM patients have high T-cell damage scores and are less sensitive to immunotherapy.
- Serum methylation level of MGMT promoter decrease during propofol and sevoflurane anesthesia in GBM patients.

## 1. Introduction

Glioblastoma (GBM) is the most common primary craniocerebral malignant tumor arising from the canceration of glial cells in the brain and spinal cord, with a high degree of malignancy and a low 5-year survival rate. At present, there are many established factors affecting its progression, including patient age, tumor grade, gene mutation, extent of surgical resection, and radiotherapy and chemotherapy [1,2]. With the development of the disease, about 90% of malignant tumor patients eventually die of tumor recurrence and metastasis [3,4]. There are many key links in the occurrence and development of malignant tumors. For surgical patients, the body is in a state of stress during the perioperative period, and surgery will induce different degrees of systemic inflammatory responses. The operation and anesthesia process will affect the function of the body’s immune system. The choice of anesthesia methods and anesthesia drugs may also affect the prognosis of patients [5–7].

Despite the relatively short duration of anesthetic exposure during glioma resection, the hemodynamic changes involved and the drugs used may also affect postoperative outcomes in glioma patients.The choice of anesthesia method will affect the prognosis of cancer. In addition, some anesthetic drugs such as propofol, dexmedetomidine and thiopental sodium may affect the malignant phenotype of tumor cells in some way [8,9]. These anesthetics are considered potential tumor suppressors or facilitators, but their effects in glioma remain to be revealed [10,11].

Cellular immunity plays an important role in the body’s anti-tumor immunity. Current research suggests that NK cells have a direct killing effect on tumor cells. In addition, T lymphocytes and various antigen-presenting cells are also involved [12]. Normally, the immune system recognizes and eliminates mutated cells. However, some tumor cells have altered surface antigens that allow them to escape the body’s immune surveillance and continue to grow and metastasize [13]. Both anesthesia and surgical stimulation can cause different degrees of immunosuppression, which further deteriorates the body’s fragile immune function against tumors [14]. Previous studies have shown that the choice of general anesthesia has a certain impact on the immune function of the body after surgery [15]. Inhalation anesthetics and some intravenous anesthetics inhibit cellular immune function, and their inhibition of NK cell and T cell function is more obvious [16–18].

In addition, pro-inflammatory cytokines such as IL-6, IL-8 and anti-inflammatory cytokines such as IL-10 play an important role in the process of the body’s immune system. The above cytokines is also closely related to the prognosis of patients with malignant tumors. Some researchers chose glioma cells from patients as research objects, treated them with sevoflurane, and analyzed related experiments. The results showed that sevoflurane can significantly increase the expression of pro-inflammatory cytokine IL-6. In addition, sevoflurane was able to increase the transcription of NF-κB factors [5,6]. In breast cancer patients, equivalent doses of propofol and sevoflurane have similar effects on immune cells such as lymphatic T cells and NK cells [19–21].

Anesthetic drugs can affect the immune function of the body, and from the current research results, sevoflurane mostly plays a negative role in this link, while the effect of propofol on immune function is relatively vague, and it is not yet possible to make a definite conclusion based on the existing evidence. In this study, the effect of propofol or sevoflurane commonly used in surgery on the malignant phenotype of GBM and the relationship with related gene expression were explored, and a new model was established to evaluate the prognosis of GBM patients with anesthetics.

## 2. Materials and methods

### 2.1. Data collection and processing

Download the gene expression profile GSE179004 from the Gene Expression Omnibus (GEO) database (https://www.ncbi.nlm.nih.gov/gds), including the corresponding clinical features (relapse status and time to relapse) [22]. The data set included two groups, the TIVA group (received propofol-remifentanil intraoperatively) and INHA group (received sevoflurane-remifentanil intraoperatively). Specimens used for gene microarray analysis were matched according to the time of anesthesia and operation, tumor location and grade, and pathological type. Gene expression data were then analyzed using weighted gene co-expression network (WGCNA) [23]. Outliers need to be removed before performing WGCNA. Then, Pearson correlation analysis was used to evaluate the relationship between gene pairs, and the results were used to construct a similarity matrix. Subsequently, WGCNA clustered genes in co-expressed modules using appropriate soft power, clustered the tree into branches by cutting using a dynamic tree-cutting algorithm, and then assigned modules to different colors for visualization.

### 2.2. Gene set enrichment analysis (GSEA)

The TCGA-GBM data were downloaded from the UCSC Xena database and clustered according to the Burlywood1 group of genes using the NMF package [24]. GSEA analysis (https://software.broadInstitute.org/gsea/index.jsp) was performed in order to identify and differentiate function between primary and recurrent tissues in GBM samples. The biological process ranking used to determine enrichment in genes was derived from the differences between the two groups.

### 2.3. Lasso model analysis

The logistic LASSO model is a shrinkage method that can actively select from a large and potentially multicollinear set of variables in the regression, resulting in a more relevant and interpretable set of predictors. LASSO performs via a continuous shrinking operation, minimizing regression coefficients in order to reduce the likelihood of overfitting [25].

### 2.4. Immune cell signature analyses

Immune cell fractions in TCGA datasets were predicted via CIBERSORT using the LM22 signature matrix with permutation count without applying quantile normalization, as directed on the website [26]. CIBERSORT outperformed other methods with respect to noise, unknown mixture content and closely related cell types. CIBERSORT should enable large-scale analysis of RNA mixtures for cellular biomarkers and therapeutic targets (http://cibersort.stanford.edu/).

### 2.5. GEPIA analyses

The online database Gene Expression Profiling Interactive Analysis (GEPIA) (http://gepia.cancer-pku.cn/index.html) was used to explore prognostic values of the critical risk score genes in GBM. The median of the group was used as the cutoff to divided the high or low expression group.

### 2.6. Detection of serum methylation level of MGMT promoter

A total of 40 patients with glioma who underwent surgical treatment were retrospectively analyzed, including 20 patients in TIVA group and 20 patients in INHA group. This study was approved by Hui Zhou Municipal Central Hospital ethics committee. All patients detected the methylation level of O6-methylguanine-DNA methyltransferase (MGMT) promoter in peripheral serum before anesthesia induction 10 min (T0), 10 min after tracheal intubation (T1), 30 min begin operation (T2), at the end of operation (T3) and three days after operation (T4) were recorded in the three groups. Take 2 mL of serum, centrifuge at 2000 r/min for 10 min after lysing red blood cells, and take the upper serum. The DNA in the sample was extracted by blood/cell/tissue genomic DNA extraction kit (TIANGEN Company), and the operation steps were carried out according to the reagent instructions. The pyrosequencing instrument (QIAGEN PyroMark Q24, USA) was used for sequencing, and the instructions of Human MGMT Gene Methylation Detection Kit (Qiagen, USA) were strictly followed. The DNA concentration is 20 ng/μl, the OD 260/280 is 1.82, the detection sensitivity is 1%(10 ng/μl DNA), and the primer type is YGAYGTTYGTAGGTTTTYGT. Results Interpretation: Four CpG sites with the highest methylation level in the promoter region of MGMT gene were selected, and the methylation level of each site was taken as quantitative data in the form of percentage, and the average of the four sites was taken as the final MGMT methylation level.

### 2.7. Statistical analyses

Kaplan–Meier curves for OS analysis were presented between different subgroups. Univariate and multivariate Cox regression analyses were used to assess the correlation between patient survival and clinicopathological characteristics and risk scores. AUC describes patient survival at 1, 3, and 5 years and is used to assess the predictive power of risk score. Both sides were considered statistically significant. Significant differences in each LM22 fraction were compared using the Mann–Whitney *U* test. Differences between two groups were evaluated with two-tailed unpaired *t*-test or Mann Whitney test. Variance analysis of repeated measurements was used to compare different time points.

## 3. Results

### 3.1. Burlywood1 group genes are significantly associated with sevoflurane-treated GBM tissue

**Figure 1.**
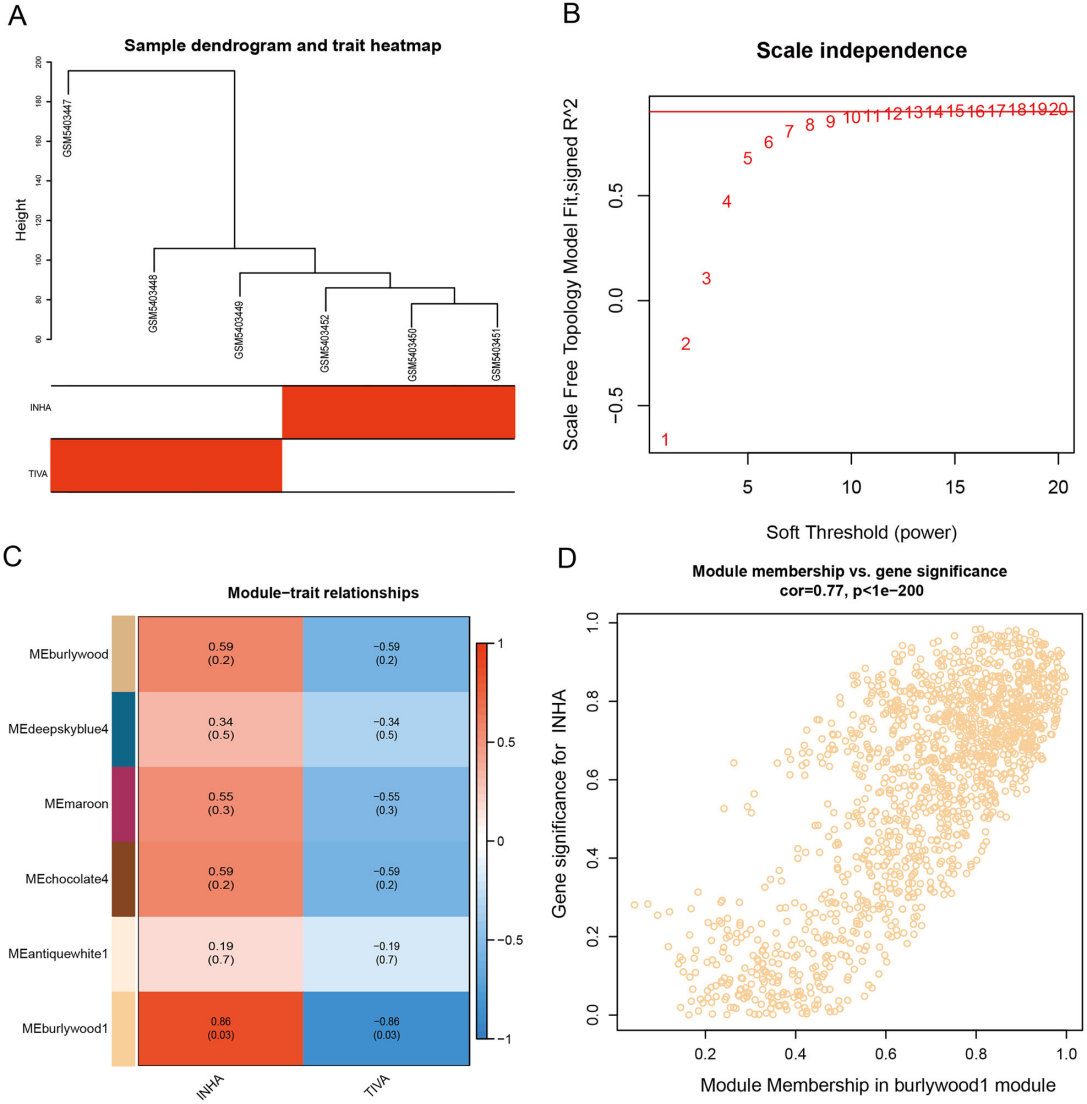
Co-expression networks were analyzed by WGCNA. (A) Sample dendrogram and trait heatmap. (B) Analysis of the scale-free index for various soft-threshold powers (β). (C) Heatmap of the correlations between the clinical traits and MEs of glioma. (D) Burlywood1 module correlation with INHA.

Co-expression modules were constructed using gene expression values in GBM samples using the WGCNA package tool. The FlashClust toolkit was used to perform cluster analysis on these samples (Figure 1(A)). One of the most critical parameters is power value, which mainly affects the independence and average connectivity of co-expression modules. When the power value is filtered out, when the power value is equal to 12, the highest degree of independence is 0.8, and the average degree of connectivity is higher. Therefore, the power values and results used to construct co-expression modules indicated that 22 distinct gene co-expression modules were identified in GBM (Figure 1(B)). Modules with co-expression patterns interaction analysis of co-expression modules are associated with specific traits was identified based on correlations between module-characterized genes and clinical features (Figure 1(C)). Finally, we plotted scatter plots of gene saliency of modules versus module membership, respectively (Figure 1(D)). The results showed that Burlywood1 group genes were significantly associated with sevoflurane-treated GBM tissues.

### 3.2. Differences in survival and progression-free survival between the two groups of patients

**Figure 2.**
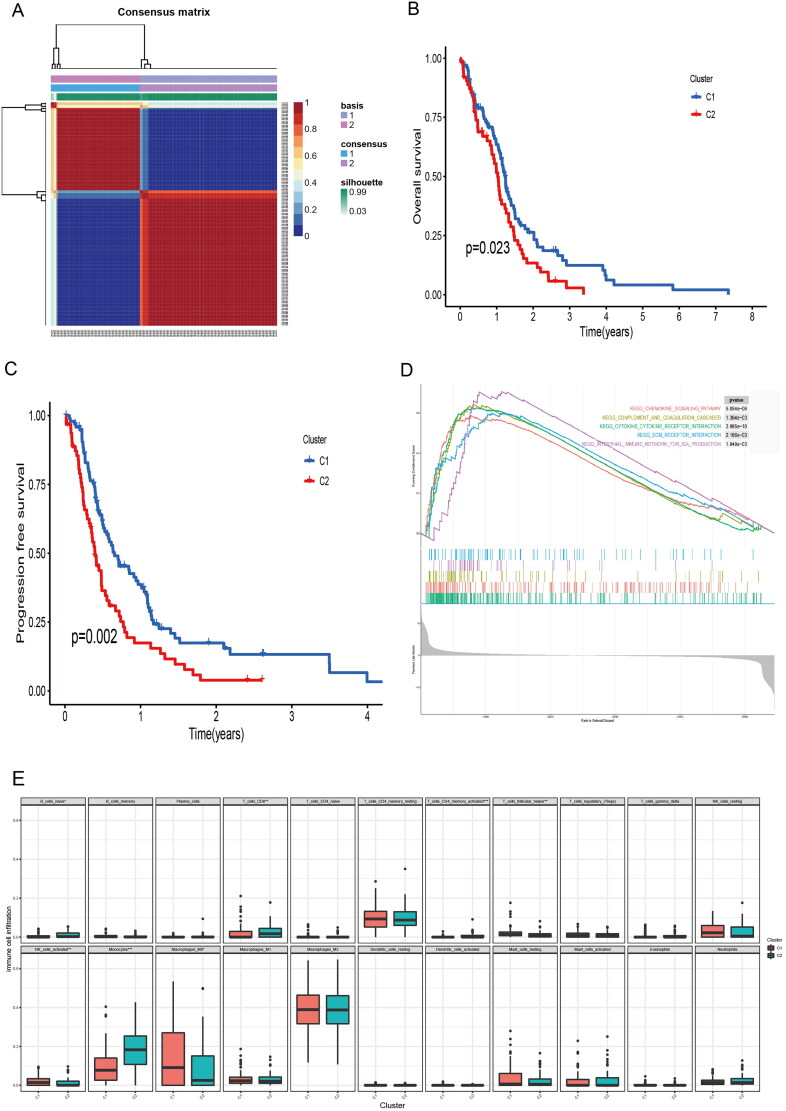
Biological characteristics of different clusters in TCGA-GBM patients. (A) Consensus matrix of TCGA-GBM patients by non-negative matrix factorization. (B) Overall survival analyses for different clusters. (C) Progression free survival analyses for different clusters. (D) Gene set enrichment analysis for different clusters.

The TCGA-GBM data were downloaded from the xena database and clustered according to the burstwood1 group genes using the NMF package (Figure 2(A)). According to the analysis results, the patients can be divided into two groups. Analysis of the overall survival and progression-free survival between the two groups of patients showed that there were significant differences in the overall survival and progression-free survival between the two groups (Figure 2(B,C)). The immune pathways of the two groups of patients were analyzed using GSEA enrichment analysis, suggesting that there were also differences in immune pathways between the two groups (Figure 2(D)).

### 3.3. PCA score was associated with TCGA-GBM prognosis

**Figure 3.**
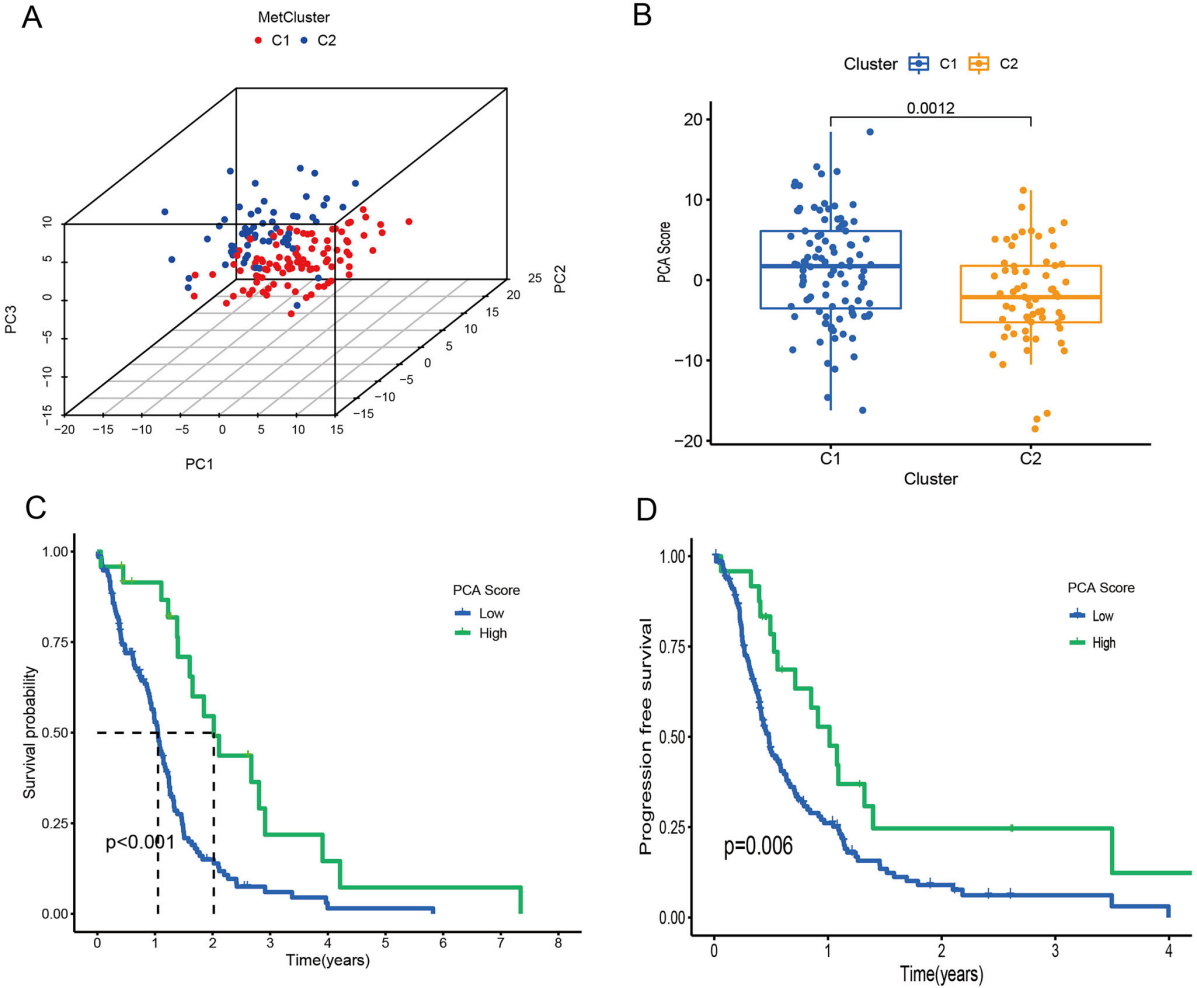
PCA scores were associated with TCGA-GBM prognosis. (A) Principal component analysis for TCGA-GBM patients. (B) PCA scores in different clusters. (C) Overall survival analyses for different clusters. (D) Progression free survival analyses for different clusters.

In biomedical applications, raw variables are often expressed in different units of measurement. PCA can be thought of as a multidimensional ellipsoid that fits the data, where each axis of the ellipsoid represents a principal component. We performed a principal component analysis of the two groups of patients from the xena database (Figure 3(A)). There were significant differences between PCA score in different clusters (Figure 3(B)). There were differences in overall survival and progression-free survival analysis between patients with high and low PCA score, and patients with low PCA score had a worse prognosis (Figure 3(C,D)).

### 3.4. The propofol and sevoflurane risk score has good predictive power

**Figure 4.**
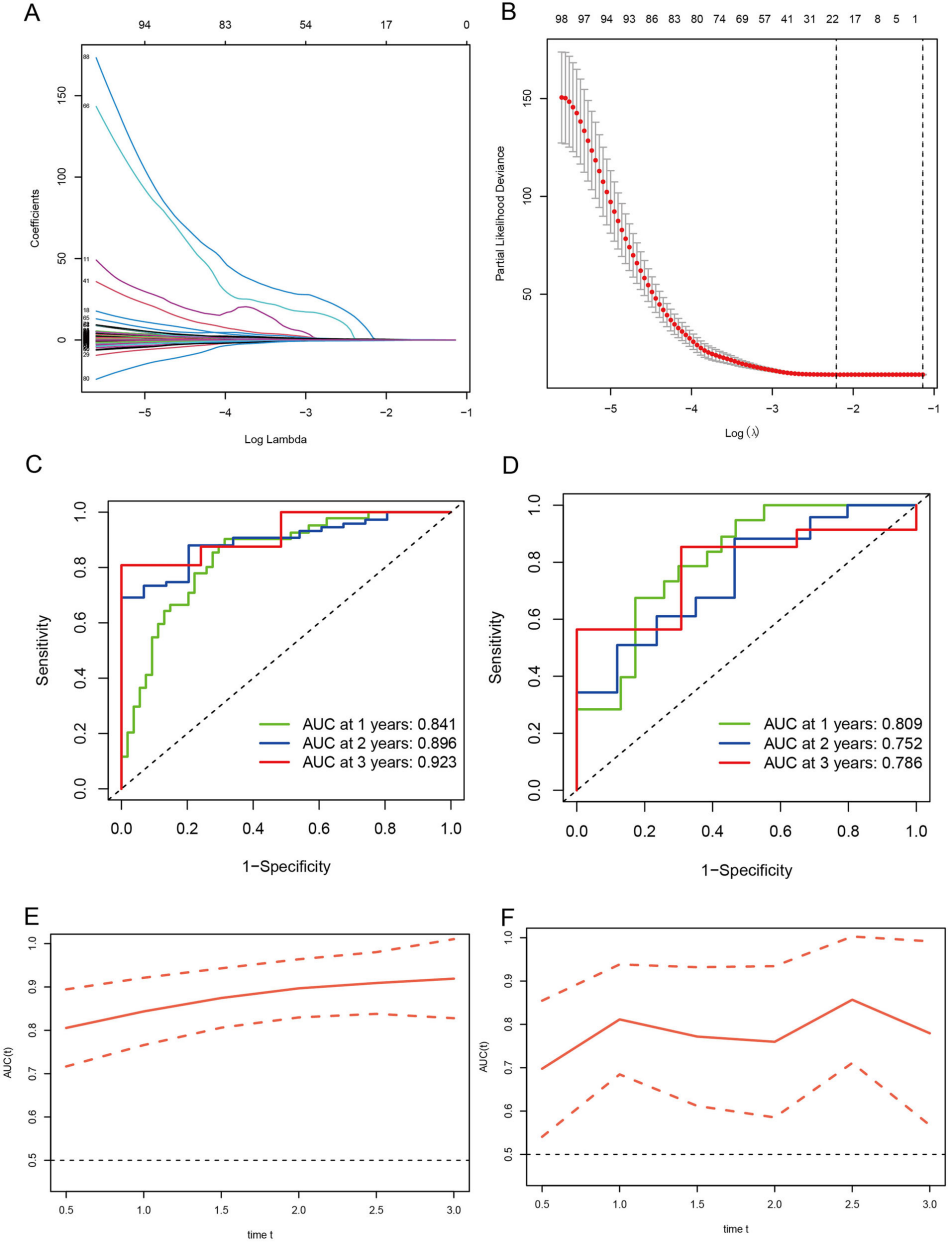
Generation of a gene expression signature to predict patient survival. (A) The 10-fold cross-validation for variable selection in the LASSO model. (B) The LASSO coefficient profile. (C) ROC curves to evaluate the predictive ability of the risk model in the train cohort. (D) ROC curves to examine the robustness of the risk model in the test cohort. (E) AUC change curves of the train cohort in 3 years. (F) AUC change curves of the test cohort in 3 years.

Logistic LASSO models can produce a more relevant and interpretable set of predictors. The LASSO prognostic model was established according to the above-mentioned differential changes, and the results showed the results of the variables included in the LASSO regression and their corresponding coefficients for different values of different parameters (Figure 4(A)). We choose a *λ* because it leads to tighter weighting, allowing us to further reduce the number of covariates than the previous *λ* (Figure 4(B)). The risk score base on the propofol and sevoflurane anesthesia related genes was calculated as follows: Risk score = SUM (0.00672 × ACOT7 + 0.41778 × GALE + 0.04479 × NUAK2 + 0.12629 × ACTA1 − 0.15987 × EEF1B22 + 0.03332 × NMNAT3 + 0.10188 × RPL39L + 0.07197 × PCDHB3 + 0.12208 × GUCA1A + 0.04046 × MICALL2 + 4.81818 × OR2F2 + 0.56677 × SLC35G5 + 0.15531 × MGMT + 0.09314 × TSPAN4 + 0.02527 × TH + 0.45407 × PDE6H − 0.40309 × USP44 + 0.01325 × NOL3 + 0.06436 × NT5M + 0.06600 × ETV4 + 0.28515 × NLRP12 + 0.01563 × RENBP).

**Figure 5.**
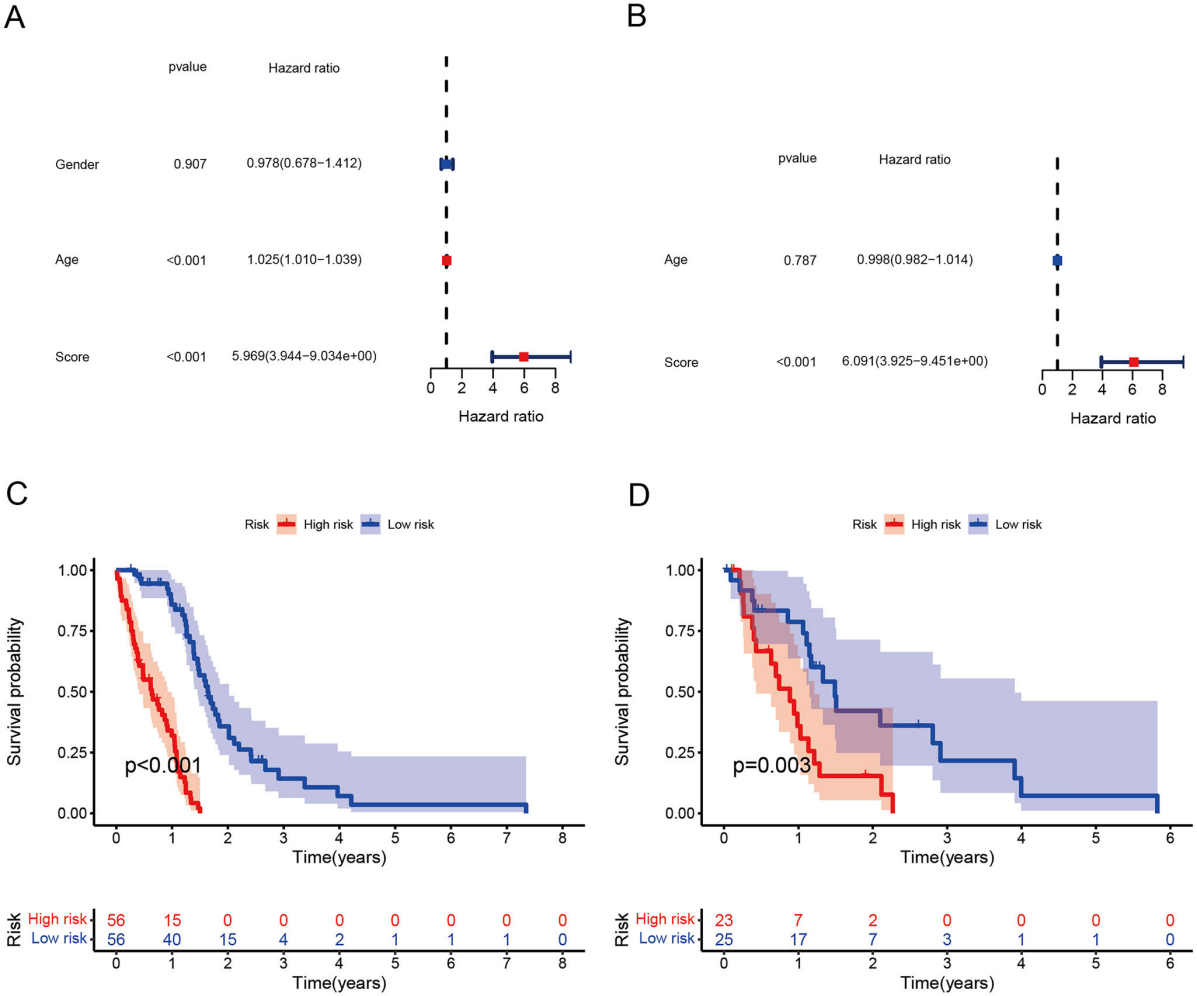
Risk score is an independent risk factor for prognosis. (A) Univariate Cox regression analyses of traits. (B) Multivariate Cox regression analyses of traits. (C) Survival analysis of risk scores in train group. (D) Survival analysis of risk scores in test group.

ROC curves were used to assess the predictive power of the risk model in the training cohort as well as to check the robustness of the risk model in the test cohort (Figure 4(C,D)). In addition, AUC curves of the 3-year training cohort and the test cohort (Figure 4(E,F)). The results show that both the training group and the validation group have good predictive ability. Furthermore, univariate and multivariate analysis indicated that risk score was an independent prognostic factor for GBM in the TCGA cohort (Figure 5(A,B)). In order to further apply the risk score to clinical prognosis prediction, we performed survival analysis on the training group and the validation group, and there was a significant difference in survival between the two groups (Figure 5(C,D)).

### 3.5. Prognostic values of risk score genes in GBM

**Figure 6.**
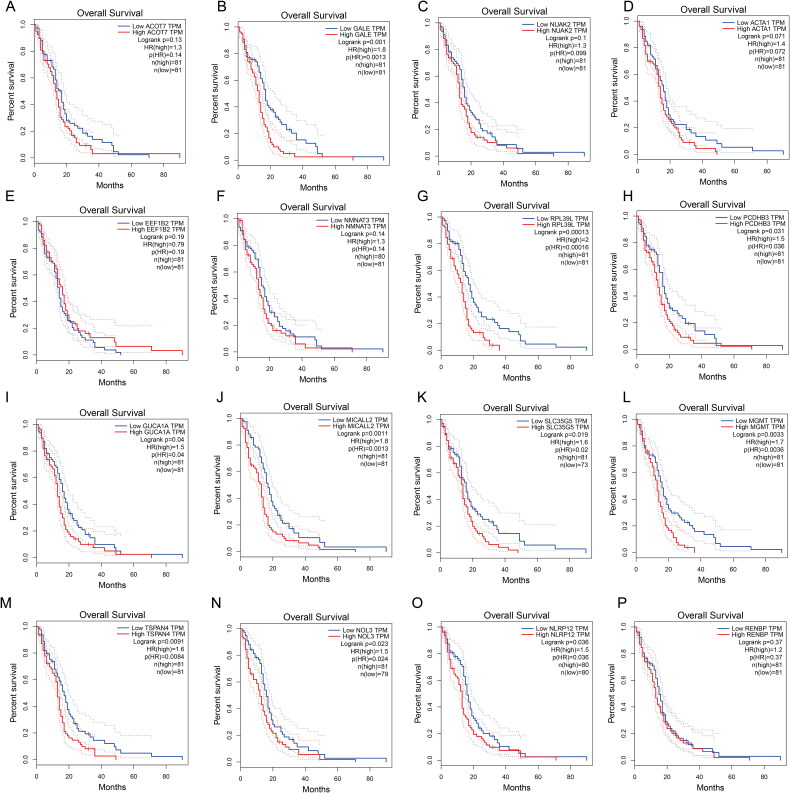
Prognostic value of the co-expression genes after propofol and sevoflurane anesthesia in GBM. (A) ACOT7; (B) GALE; (C) NUAK2; (D) ACTA1; (E) EEF1B22; (F) NMNAT3; (G) RPL39L; (H) PCDHB3; (I) GUCA1A; (J) MICALL2; (K) SLC35G5; (L) MGMT; (M) TSPAN4; (N) NOL3; (O) NLRP12; (P) RENBP.

To further explore the prognostic value of risk score genes in GBM, we conducted the Survival Plots to identify the gene expression level and the prognostic value in GBM. As showed in Figure 6, we found higher GALE, RPL39L, PCDHB3, GUCA1A, MICALL2, SLC35G5, MGMT, TSPAN4, NOL3 and NLRP12 expression are correlated with poor overall survival in GBM patients (Figure 6(B,G–O)). However, the expression level of ACOT7, NUAK2, ACTA1, EEF1B22, NMNAT3 and RENBP had no correlation with the overall survival of GBM patients (Figure 6(A,C–F,P)). These interesting result further confirm the propofol and sevoflurane anesthesia will impact the gene expression and closely correlated with prognosis in GBM.

### 3.6. Correlation of risk scores with clinical characteristics

**Figure 7.**
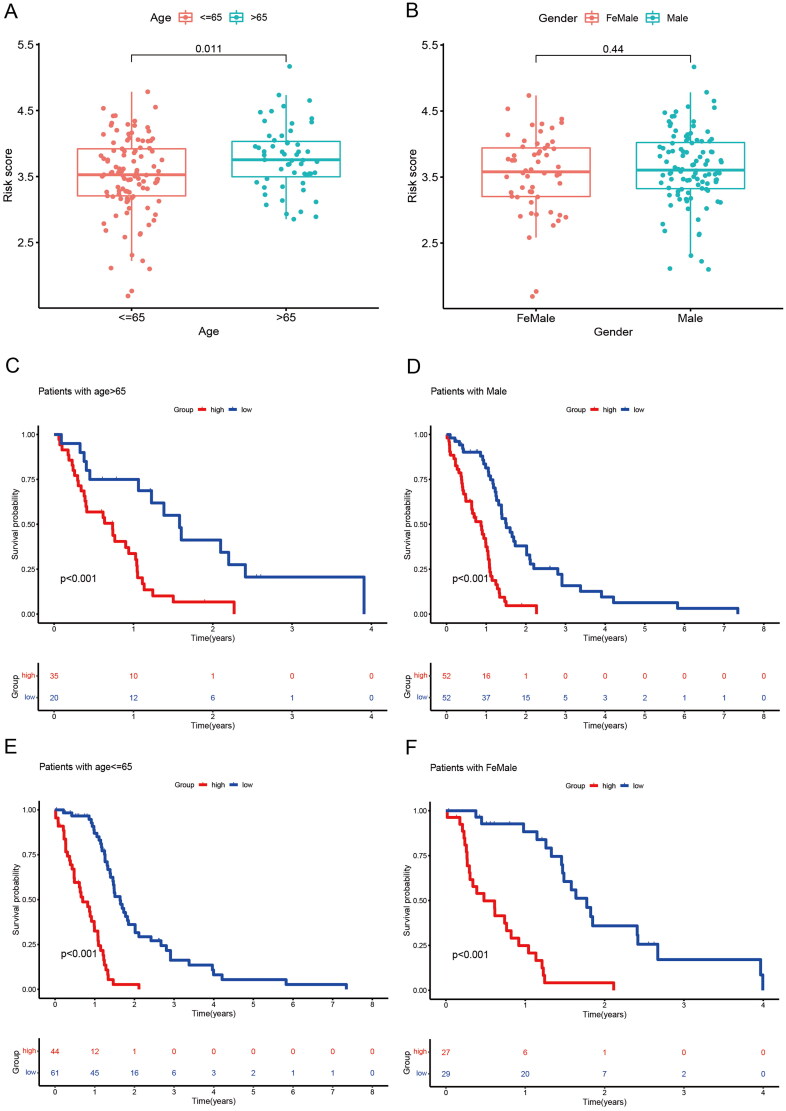
Correlation between risk score and clinical traits and subgroup survival analysis. (A) Correlation between risk score and age. (B) Correlation between risk score and Gender. (C) Survival analysis of risk scores in patients with age > 65. (D) Survival analysis of risk scores in patients with male. (E) Survival analysis of risk scores in patients with age< =65. (F) Survival analysis of risk scores in patients with female.

We assessed the prognostic value of risk score for associated genes in different subgroups of GBM patients. GBM patients older than 65 years had higher risk score than those younger than 65 years (Figure 7(A)). There were no differences between GBM gender subgroups (Figure 7(B))). Next, we further analyzed the predictive value of risk scores across different clinical features. The high-risk group had a poor prognosis regardless of age and gender (Figure 7(C–F)).

### 3.7. Sensitivity of patients in the prognostic risk model group to immunotherapy

**Figure 8.**
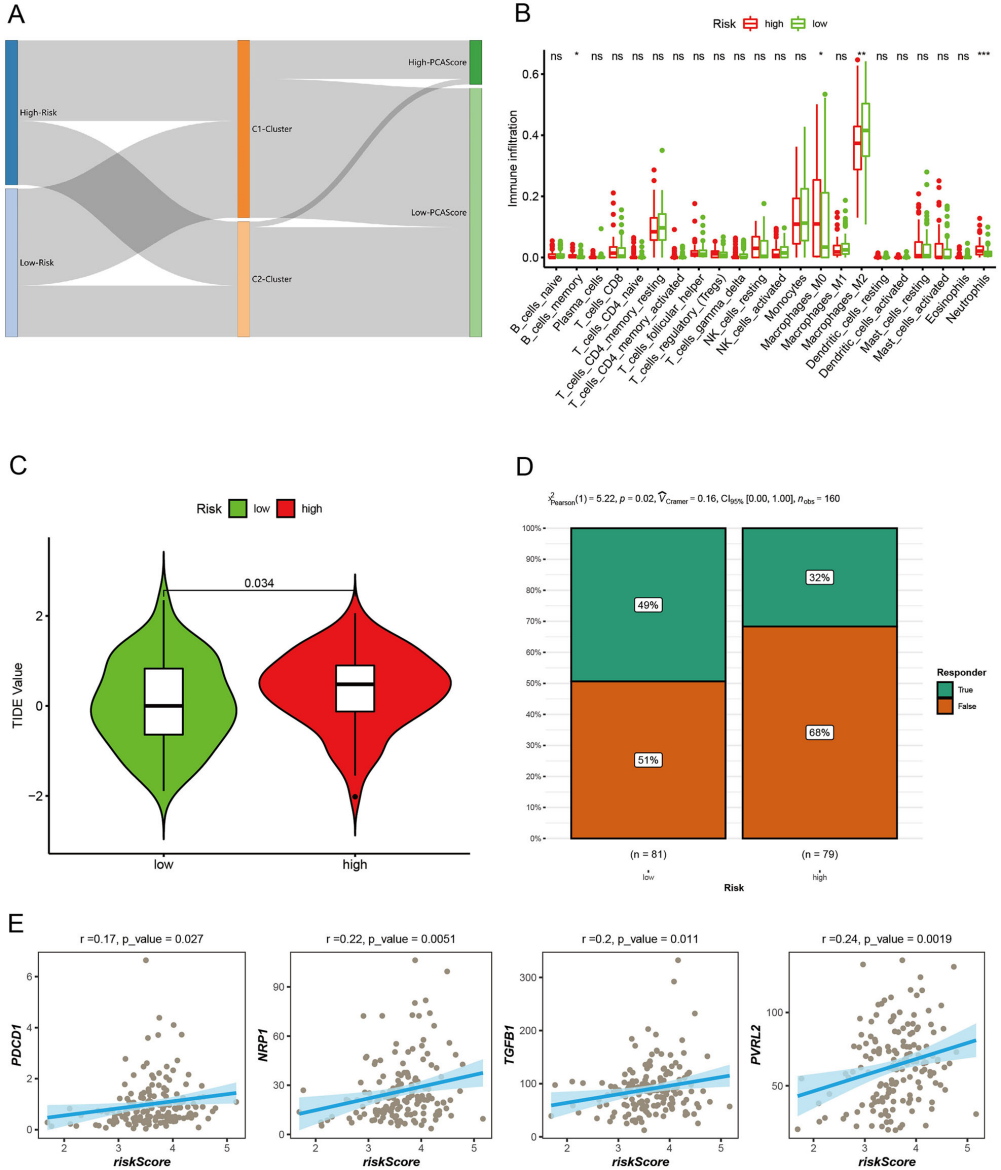
Immune microenvironment and immunotherapy. (A) Alluvial diagram showing the changes of risk score, cluster, and PCA score. (B) Differences of immune cell infiltration in different risk groups by CIBERSORT. (C) The TIDE values in high and low risk score groups. (D) The likelihood of the clinical response to antiPD1 and anti-CTLA4 therapy for high and low risk score patients from the TCGA cohorts. True represents immunotherapy responders, while false represents immunotherapy nonresponders. (E) Correlation analysis of risk scores and immune-related markers.

We further analyzed the relationship between the prognostic risk score of the two groups of patients and the Burlywood1 group of patients, as well as the PCA score-related clusters, and visualized them using an alluvial plot (Figure 8(A)). The results showed that most of the low-risk patients belonged to C1, and most of the patients with high PCA score also belonged to C1. To determine the relative abundance of tumor-infiltrating immune cells (TIICs) in GBM samples, the degree of infiltration of TIICs was estimated using the CIBERSORT algorithm. There was a statistically significant difference in immune cell infiltration between the two groups, with higher memory B cells, M0 macrophages, and neutrophils in high-risk patients (Figure 8(B)). Subsequently, TIDE (Tumor Immune Dysfunction and Exclusion) assessed the relationship between GBM risk score and immunotherapy (Figure 8(C)). The chi-square plot showed that 49% of responders in the low-risk group were responders and 51% were ineffective in TIDE; in the high-risk group, 68% of responders were ineffective and 32% were effective (Figure 8(D)). Finally, the correlation analysis of risk score and immune-related markers was assessed (Figure 8(E)). High-risk patients have high T-cell damage score and are less sensitive to immunotherapy.

### 3.8. Serum methylation level of MGMT promoter change during propofol and sevoflurane anesthesia

**Figure 9.**
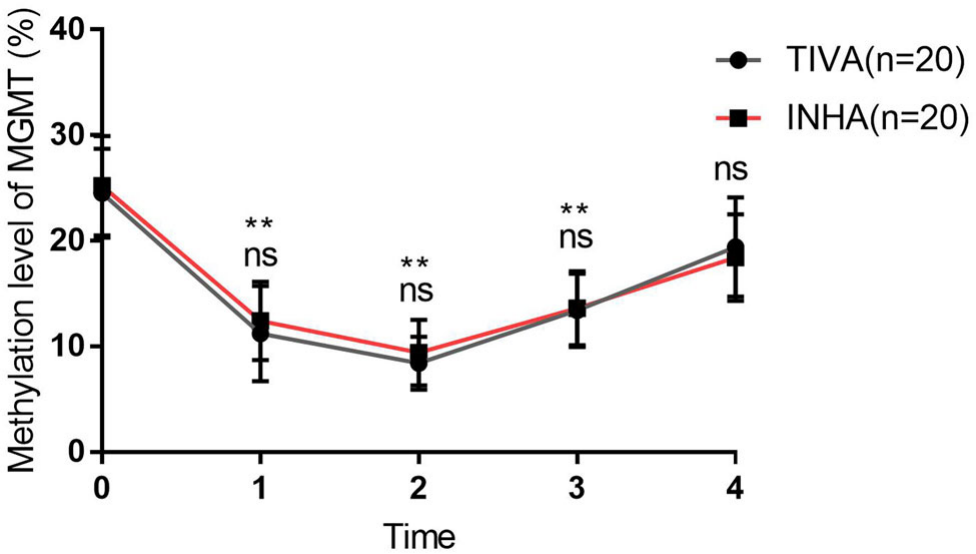
Serum methylation level of MGMT promoter before and after propofol and sevoflurane anesthesia. The serum methylation level of MGMT promoter in TIVA group and INHA group. (ns: no significant difference).

As we found many critical co-expression genes in the GBM sample during propofol and sevoflurane anesthesia. MGMT methylation is one of the independent predictors of good prognosis of glioma patients. At last, we detect the serum methylation level of MGMT gene promoter before and after operation of patients received propofol-remifentanil (TIVA group) or sevoflurane-remifentanil (INHA group) anesthesia. The base information of the patients were listed in Supplemental Table 1. And the serum methylation level of MGMT gene promoter in T0 has no correlation with age, gender, tumor diameter and WHO classification in GBM patients (Table 1). There is no significant difference of serum methylation level between TIVA and INHA groups in T0, T1, T2, T3 and T4. However, the serum methylation level was significantly decreased in T1, T2 and T3 (Figure 9). This result suggests serum methylation level of MGMT promoter decrease during propofol and sevoflurane anesthesia.

**Table 1.** Correlations between methylation level of MGMT promoter and clinicopathological characteristics in GBM.

Parameters	No	Methylation level of MGMT promoter		*p*-Value
		Low (<20%) (*n* = 19)	High (≥20%) (*n* = 21)	
Age (years)				0.525
<50	22	9	13	
≥50	18	10	8	
Gender				0.488
Male	29	15	14	
Female	11	4	7	
Tumor diameter (cm)				0.538
<4	17	7	10	
≥4	23	12	11	
WHO (advanced/low)				0.689
Advanced	33	15	18	
Low	7	4	3	

## 4. Discussion

Sustained tumor growth and metastasis are the main causes of death in patients with malignant tumors. At the same time, in order to adapt to the internal hypoxic metabolic environment, tumor cells change the expression of various factors, among which the up-regulation of the expression of hypoxia-inducible factor family is crucial [27,28]. Sevoflurane have been reported to significantly inhibit tumor growth, but some study also reports Sevoflurane does not promote the colony-forming ability of human mesenchymal glioblastoma stem cells *in vitro* [29]. Moreover, sevoflurane nor propofol used as anesthetics modulate MMP-2 and MMP-9 concentrations and activities during non-oncological, non-vascular elective surgery [30]. The immune function of patients with GBM is poor, and the immune function can deteriorate under the stimulation of surgery and anesthesia. Clinical studies have shown that the immune function of the body can lead to different immunosuppression due to different anesthetic drugs, and the immunosuppression can last until surgery, and during this period, patients are prone to perioperative complications, such as infection and hypostatic pneumonia. These complications can further worsen immune function [30]. Therefore, the selection of appropriate anesthesia drugs is an important factor to ensure smooth operation and postoperative recovery. In this study, propofol or sevoflurane commonly used in surgery on the malignant phenotype of GBM and the relationship with related gene expression were explored, and a new model was established to evaluate the prognosis of GBM patients with anesthetics.

Propofol is a widely used intravenous anesthesia drug with a rapid onset of action and can be used for anesthesia induction and anesthesia maintenance. Some studies have found that propofol used in neurosurgery can play a certain role in brain tissue protection, but excessive use may lead to cardiac or respiratory depression [31]. Propofol not only has obvious sedative effects, but also protects organ function, inhibits platelets and regulates the release of immune factors [32]. Sevoflurane is an inhaled anesthetic drug, mainly metabolized by the respiratory tract and lungs. It has the characteristics of rapid induction of anesthesia, shorten recovery time, and no toxic side effects. It often uses in clinical induction of respiratory anesthesia [33]. Studies have found that sevoflurane can inhibit the proliferation and migration of cancer cells *in vitro*. Therefore, anesthesia methods or anesthetic drugs may have a certain impact on postoperative recurrence, survival and outcome of tumor patients. The use of anesthetic drugs or anesthesia methods that have a certain inhibitory effect on tumor cell proliferation and invasion may reduce tumor cell metastasis and dissemination during surgery, thereby reducing the probability of tumor recurrence or metastasis [34].

When the number and function of T lymphocyte subsets in the body change, immune dysfunction will occur. Selective differentiation of Th cells is also related to tumor prognosis. Th1 cells differentiated from them secrete cytokines such as IL-2 and IL-12, activate antigen-presenting cells, enhance the activity of NK cells, and play an anti-tumor effect. The Th2 cell subset mainly regulates the humoral immune process, inhibits the activity of NK cells and Th1 cell subsets, and is beneficial to the proliferation and metastasis of tumor cells [35,36]. Studies have shown that propofol has a stable anesthetic effect and obvious sedative effect, which can significantly reduce the agitation in patients during surgery. At the same time, it can protect damaged neurons and ischemic organs, thereby reducing platelet aggregation [37]. It improves the hemodynamics of patients, and at the same time, it has a certain regulatory effect on immune function, which is conducive to the recovery of patients’ immune function and promotes the recovery of the body [38]. The mechanism by which propofol can inhibit the malignant potential of cancer cells may be related to down-regulation of PD-L1 expression [39]. Propofol can inhibit the metabolic process of tumor cells and promote their apoptosis.

The idea that general anesthetics may alter the prognosis of patients with malignant tumors has been widely recognized. Our experiments showed that the screened burstwood1 group was significantly associated with sevoflurane-treated GBM tissue. The patients were divided into two groups, and there were differences in survival and progression-free survival between the two groups. GSEA enrichment analysis indicated that there were also differences in immune pathways between the two groups. Through differential analysis, 22 independent prognostic differential genes were screened and incorporated into the random forest prognostic model. LASSO prognostic model analysis showed that both the training group and the validation group had good predictive ability. Univariate and multivariate analysis, the two groups were divided into high and low groups according to the scores, and the survival of the two groups was significantly different. The risk score was strongly correlated with the age of the patients, but not with the sex of the patients. The final results analyzed the differential responses of high and low risk groups to immunotherapy, indicating that sevoflurane signature genes were consistent in the classification of gliomas. High-risk patients have high T-cell damage scores and are less sensitive to immunotherapy.There are multiple correlations between anesthetic drugs and GBM clinicopathology in this study, but the specific mechanism is unclear. In the future, *in vitro* and *in vivo* experiments and larger cohorts are still needed to conduct in-depth research, and further establish grading standards to promote better clinical research.

At last, as MGMT is a critical co-expression gene in our model. Previous studies had reported that MGMT expression is a critical biomarker for GBM [40,41]. And the MGMT methylation is one of the independent predictors of good prognosis of glioma patients [42]. Therefore, our study further detects the serum methylation level of MGMT promoter change during propofol and sevoflurane anesthesia. We found the serum methylation level was significantly decreased in during the intervention of propofol and sevoflurane anesthesia. In recent years, a growing body of literature suggests that anesthesia-induced long-term changes in gene transcription and functional deficits in learning and behavior later in life are mediated via epigenetic modifications [43]. This study firstly found that serum methylation level of MGMT promoter decrease during propofol and sevoflurane anesthesia. As the less clinical sample size, and there are many influencing factors of different patients, so it still needs a larger sample size evaluation the influence of propofol and sevoflurane on the methylation level of MGMT promoter in GBM patients.

## 5. Conclusions

In this study, bioinformatics methods were used to analyze multi-omics data of GBM samples in public databases, and it was found that the propofol and sevoflurane anesthesia associated impact the gene expression of GBM, include the methylation level of MGMT promoter. Propofol and sevoflurane anesthesia-based risk score prognostic model, which has good prognostic power and is an independent prognostic factor in GBM patients. Therefore, this model can be used as a new biomarker for judging the prognosis of GBM patients.

## Supplementary Material

Supplemental Material

## Data Availability

All analyzed data are included in this published article. The original data are available upon reasonable request to the corresponding author.
