# Supplementary material for: Co-expression prognostic-related genes signature base on propofol and sevoflurane anesthesia predict prognosis and immunotherapy response in glioblastoma
Source: Ann Med. 2023 Mar 1;55(1):778–92. doi: 10.1080/07853890.2023.2171109 (PMC9979995; doi:10.1080/07853890.2023.2171109)
Supplement: Supplemental Material [file IANN_A_2171109_SM0996.docx]

Supplemental table 1. General information of patients

| Index | TIVA (n=20) | INHA (n=20) | *P* |
| --- | --- | --- | --- |
| Gender (male/female) | 15/5 | 14/6 | >0.05 |
| Age (year) | 49±7 | 50±2 | >0.05 |
| ASA (Ⅱ/Ⅲ) | 18/2 | 17/3 | >0.05 |
| Tumor diameter (cm) | 4.11±1.83 | 4.26±1.62 | >0.05 |
| WHO (advanced/low) | 16/4 | 17/3 | >0.05 |
| Anesthesia time | 382±46 | 357±56 | >0.05 |
